# The Many Organisational Factors Relevant to Planning Change in Emergency Care Departments: A Qualitative Study to Inform a Cluster Randomised Controlled Trial Aiming to Improve the Management of Patients with Mild Traumatic Brain Injuries

**DOI:** 10.1371/journal.pone.0148091

**Published:** 2016-02-04

**Authors:** Marije Bosch, Emma J. Tavender, Sue E. Brennan, Jonathan Knott, Russell L. Gruen, Sally E. Green

**Affiliations:** 1 Department of Surgery, Central Clinical School, Monash University, Melbourne, Australia; 2 National Trauma Research Institute, The Alfred, Monash University, Melbourne, Australia; 3 School of Public Health and Preventive Medicine, Monash University, Melbourne, Australia; 4 Melbourne Medical School, The University of Melbourne, Melbourne, Australia; 5 Department of Emergency Medicine, Royal Melbourne Hospital, Melbourne, Australia; 6 The Alfred Trauma Service, The Alfred Hospital, Melbourne, Australia; 7 Lee Kong Chian School of Medicine, Nanyang Technological University, Singapore, Singapore; Azienda Ospedaliero-Universitaria Careggi, ITALY

## Abstract

**Background:**

The Neurotrauma Evidence Translation (NET) Trial aims to design and evaluate the effectiveness of a targeted theory-and evidence-informed intervention to increase the uptake of evidence-based recommended practices for the management of patients who present to an emergency department (ED) with mild head injuries. When designing interventions to bring about change in organisational settings such as the ED, it is important to understand the impact of the context to ensure successful implementation of practice change. Few studies explicitly use organisational theory to study which factors are likely to be most important to address when planning change processes in the ED. Yet, this setting may have a unique set of organisational pressures that need to be taken into account when implementing new clinical practices. This paper aims to provide an in depth analysis of the organisational context in which ED management of mild head injuries and implementation of new practices occurs, drawing upon organisational level theory.

**Methods:**

Semi-structured interviews were conducted with ED staff in Australia. The interviews explored the organisational context in relation to change and organisational factors influencing the management of patients presenting with mild head injuries. Two researchers coded the interview transcripts using thematic content analysis. The “model of diffusion in service organisations” was used to guide analyses and organisation of the results.

**Results:**

Nine directors, 20 doctors and 13 nurses of 13 hospitals were interviewed. With regard to characteristics of the innovation (i.e. the recommended practices) the most important factor was whether they were perceived as being in line with values and needs. Tension for change (the degree to which stakeholders perceive the current situation as intolerable or needing change) was relatively low for managing acute mild head injury symptoms, and mixed for managing longer-term symptoms (higher change commitment, but relatively low change efficacy). Regarding implementation processes, the importance of (visible) senior leadership for all professions involved was identified as a critical factor. An unpredictable and hectic environment brings challenges in creating an environment in which team-based and organisational learning can thrive (system antecedents for innovation). In addition, the position of the ED as the entry-point of the hospital points to the relevance of securing buy-in from other units.

**Conclusions:**

We identified several organisational factors relevant to realising change in ED management of patients who present with mild head injuries. These factors will inform the intervention design and process evaluation in a trial evaluating the effectiveness of our implementation intervention.

## Introduction

Mild head injuries, caused by external forces to the head (such as falls, motor vehicle accidents, or assaults) [1], are a frequent cause for emergency department (ED) presentations worldwide [2]. The challenge for the emergency physician is to identify which patients with a head injury have an actual traumatic brain injury (TBI) requiring further management, and which patients can safely be sent home. Patients with subtle symptoms and signs can still progress to adverse outcomes [3]. In addition, up to 15% of patients diagnosed with mild traumatic brain injury (mTBI) experience persistent disabling problems [4,5]. Several evidence-based clinical practice guidelines for the management of this patient group exist [6], yet research shows inconsistent implementation of recommended practices [7–9]. As the ED is often the only point of medical contact for this patient group, optimising ED care has the potential to optimise outcomes for these patients. This paper is part of a larger study (The Neurotrauma Evidence Translation (NET) Trial) aiming to design and evaluate a targeted theory- and evidence-informed intervention to improve adoption of evidence-based recommended practices (see Table 1) for the management of mild head injured patients in Australian EDs [10,11]. A description of the relevance of these recommendations to the management of this patient group, and evidence regarding gaps in practice can be found in the trial protocol [11]

**Table 1:** Four key recommended practices.

Post-traumatic amnesia should be prospectively assessed in the emergency department using a validated tool.
Guideline-developed criteria or clinical decision rules should be used to determine the appropriate use and timing of CT imaging.
Verbal and written information should be provided on discharge.
Brief, routine follow-up consisting of advice, education and reassurance should be provided.

Interventions aiming to bring about change are likely to be most effective if they address the most important determinants of practice for improvement in the targeted setting [12]. Implementation studies traditionally have tended to focus predominantly on individual professionals [13]. However, successful individual adoption is only one component of spread of complex innovations in organisations [14]. For example, a study looking at factors influencing the uptake of metered dose inhalers with spacer devices for treatment of asthma in paediatric emergency departments [15] shows that buy-in at an individual level does not guarantee adoption and incorporation of the device into routine practice. Therefore, when designing interventions aiming to bring about change in organisational settings, to ensure successful implementation of practice changes, it is important to understand the impact of the context on adoption decisions and (current) practices [16–20].

Some previous studies evaluated factors influencing implementation of guidelines in ED settings in relation to other conditions, e.g. [15,21–24]. For example, Meurer et al [21] studied barriers to thrombolytic use in acute stroke care and identified environmental factors (e.g., availability of intensive care units, ED crowding, pharmacy or radiology), patient factors (e.g., failure to recognize symptoms, preference to arrive via car instead of ambulance), and guideline factors (issues with the structure or content of guidelines in general) as ‘external’ (non-physician) factors. We were not aware of any studies reporting on barriers for the implementation of guidelines regarding managing mTBI patients in the ED, and it has been shown that the medical condition or topic of the guideline influences its uptake [25]. In addition, although the use and testing of theory-driven models of change from a range of scientific disciplines has been recommended for enhancing implementation efforts in ED settings [26,27], few studies explicitly use organisational theory to study which organisational factors are likely to be most important to address when planning change processes in the ED [28]. Yet, the ED setting may present a unique combination of organisational characteristics [29,30] influencing change and the adoption of new practices [31]. This paper aims to provide an in depth analysis of the organisational context in which ED management of mTBI and implementation of new practices occurs, drawing upon organisational level theory [20]. In a related paper, we explore individual level factors (e.g. knowledge, skills) likely to influence adoption of specific evidence-based practices for the management of mTBI in Australian EDs [32]. Findings from both studies will inform the design and delivery of an intervention that is going to be implemented as part of the NET-Trial. Given the wide variety of factors that may influence practice change, basing interventions on different theoretical assumptions may prevent overlooking important factors [33] and will ultimately help to understand how and why change is achieved.

## Methods

### Study design

Qualitative study using in-depth semi-structured interviews.

### Participants

Participants were staff working in 24hr-hospital EDs within the Australian State of Victoria. These include medical doctors, registered nurses, nurse practitioners, and ED directors. To ensure maximum variation, we aimed to recruit a stratified purposeful sample [34], including small, medium and large as well as metropolitan, inner and outer regional EDs [35]. The Australian Standard Geographical Classification-Remoteness Areas (ASGC-RA) system was used to group hospitals in terms of remoteness i.e. the physical distance of a location from the nearest urban centre [36].

### Procedure

Hospitals were identified through a Government Health Information website. ED directors received an invitation letter, explanatory statement and consent form. They were asked to indicate whether they (or a delegate e.g. deputy director) would be willing to be interviewed, and/or forward copies of the documentation to relevant staff (including those typically involved in change management or quality improvement) on behalf of the research team. Participants opted-in to the study and provided written informed consent to participate through completion of the consent form. Single face-to-face interviews were conducted at a time and location nominated by the participants. The interviews were undertaken by two researchers (ET, MB), who took turns in leading topics discussed. This allowed the other researcher to concentrate on listening, asking clarifying questions, and thinking about the questions that needed further exploration. The researchers had experience in evidence-based medicine and qualitative research methods with knowledge of the clinical field and in-depth knowledge of the project. Interviews were audiotaped and transcribed verbatim. Checked transcripts were imported into NVIVO 8 (QSR International Pty Ltd, Australia) to manage the data and facilitate the analysis. The date of the interview was added to the transcripts, allowing ‘tracking’ and development of the coding framework.

#### Interview content

Separate interview guides were developed for nursing and medical staff and ED directors (see S1 File). The interviews explored the organisational context in relation to change (e.g. current and successful change management practices) and organisational factors influencing the management of patients presenting with mild head injuries. To inform interview questions, selected literature reviews regarding planning and organising change in health care organisations (e.g. [14,37–40]) were used to identify theoretical perspectives likely to be relevant in this particular setting. Theories were selected by a multi-disciplinary team, including an ED doctor and investigators with expertise in implementation research and organisational change literature, based on a discussion around perceived relevance of theories for this particular setting. The “model of diffusion in service organisations”, included in the review from Greenhalgh and colleagues, [14], was used to guide analyses and organisation of the results. This model is “meant as a memory aide for considering the different aspects of a complex situation and their many interactions” [14], and was developed through an extensive systematic review covering 13 research areas in various disciplines (e.g. sociology, psychology, organisation and management) and identifies the main domains or areas in which factors influencing uptake and implementation of interventions in organisations are found. For the purposes of this paper, we present results under five broad domains: 1) the innovation (i.e. the evidence-based recommendations for the management of mTBI); 2) system readiness for innovation; 3) implementation processes (including linkage, assimilation and communication and influence); 4) system antecedents for innovation; and 5) outer context). Factors that fall under the domain “adopter” (e.g. individual’s skills or learning styles) are explored in depth in the related paper [32]. Table 2 shows the theoretical perspectives and potential influencing factors that were considered particularly pertinent to the aims of our study, grouped by domain.

**Table 2:** Domains, theoretical perspectives and influencing factors.

Domains */ theoretical perspectives on…*	Influencing factors	Key refs
**The innovation**		
*… diffusion of innovations*	Characteristics of an innovation (in our case this refers to characteristics of the evidence-based recommendations for the management of mTBI) that may influence its uptake, such as clear unambiguous advantage; compatibility with values and needs; low complexity; trialability; observability; potential for reinvention; and nature of knowledge required (eg tacit or explicit)	[14,41,42]
**System readiness for innovation**		
*… readiness for change*	Influencing factors such as system readiness for change / tension for change (the degree to which stakeholders perceive the current situation as intolerable or needing change) / system—intervention fit (e.g. whether the intervention meets an identified need and whether it aligns with organisational priorities)	[14,43–45]
**Implementation processes**		
*… communication*, *leadership and social network and influence*	Factors such as network structure, types of dissemination, communication and influence, social networks, formal and informal leaders	[46–51]
**System antecedents for innovation**		
*… organisational culture*	Perceptions of organisational values and characteristics such as organisational culture in relation to change; receptivity for change; organisational history of change	[14,52,53]
*… innovative organisations*	Structural organisational characteristics that may influence the uptake of an intervention such as human and physical resources; organisational slack; size; differentiation	[54,55]
*… organisational learning & knowledge management*	Characteristics such as the capacity to create, acquire and transfer information, single or double loop learning, enablement of knowledge sharing via networks; team climate for innovation	[56–62]
**Outer context**		
*… integrated care / cross-unit collaboration / quality management*	Factors in the broader hospital such as the presence or absence of cross-unit policies, hospital wide key performance indicators, and decision-making structures	[63–65]
*…wider political or economic context*, *e*.*g*. *regulation*, *reimbursement*	Factors related to the wider healthcare system such as presence or absence of mandatory policy regulations at national or state level, reimbursement systems and care paths that span the entire patient journey	[66–68]

#### Analysis

Data were analysed using an iterative process. Two researchers (MB/ET) independently open coded transcript text to domains. Text fragments relevant to more than one domain were cross-indexed. The first author of this paper (MB) created codes for emerging factors and categories of factors (i.e. themes) within theoretical perspectives. A third author with knowledge of the organisational science, management and innovations literature (SB) read a subset of the transcripts and independently identified factors. The final coding framework was developed after comparing, discussing and agreeing on the codes (MB/SB). We produced an audit trail by keeping a record of coding decisions [69]. Factors were considered important according to saliency analysis i.e. by explicitly assessing from the data which code recurs, or is considered highly important to the researchers or participants, or has both attributes [70]. Quotations were used from the transcripts for illustration.

#### Ethics

Ethics approval was obtained from the Monash University Human Research Ethics Committee (MUHREC)–Project Number: CF10/2343–2010001338.

## Results

Interviews were conducted over a seven month period (November 2010 to May 2011). They were predominantly held face-to-face, however some were held by telephone. Thematic saturation was reached after 42 interviews with participants from 13 hospitals (eight major city, four inner regional and one outer regional)[71]. Nine ED directors, 14 senior doctors (consultants/medical directors), six junior doctors (registrars), and 13 nurses (including nurse unit managers, bed managers, and nurse educators) participated.

Table 3 summarises the main findings, which are described in more detail below. S2 File lists the influencing factors, arranged by domain, including illustrative quotations.

**Table 3:** Summary of main findings.

Main influencing factors
**The innovation**
• Guideline-based intervention low compatibility with medical culture; good compatibility with nursing culture
• Potential for reinvention (e.g. to reflect available resources)
• Changes need to be observable to keep momentum / commitment
• Needs clear, unambiguous advantage over current practice
• High complexity of cross-unit change
**System readiness for innovation**
• Relatively low tension for change / perceptions of collective change commitment for “acute part of management” (generally not perceived as in need of change)
• Mixed tension for change for management of longer-term symptoms (higher change commitment, but relatively low change efficacy)
• Management driven agenda perceived to be very time-focused and not necessarily focused on high quality management from patient perspective
**Implementation processes**
• Different professions have own systems in place for organising and communicating changes
• Influence within social networks, not across (particularly in medical professions)
• Visible multi-disciplinary leadership, use of ‘stable forces’ required
• Respected (informal or formal) leaders
**System antecedents for innovation**
• High staff turnover rates generally perceived to hamper implementation due to constant loss of tacit knowledge
• Little organisational slack, stretched environment
• ED perceived to be open to change in general, positive culture in relation to change (relatively positive history of change)
• Stretched and hectic ED environment not conducive to learning and reflection
• Constantly changing team-structure bring challenges to team-based learning
• Lack of routine monitoring and feedback (as well as systems to support this); predominantly reactive approaches to problem solving
• Coordination between various quality systems still very manual
**Outer context**
• Being subspecialty at the entry-point of the hospital means many specialties have requests with respect to the management if they were to admit patients under their care
• Absence of agreed cross-unit pathways / protocols
• Agreement between different specialties generally difficult to organise
• Accountability metrics very finance driven
• Financial systems focus on local costs; no entire patient care journey through the system; perceived absence of follow up facilities

### The innovation

#### Characteristics that are perceived to influence the uptake of the innovation

Given our recommended practices are guideline-based we explored attitudes in relation to use of guidelines and clinical pathways in the ED. Compatibility with values and perceived needs appeared the most important influencing factor. In general participants agreed that the use of guidelines fits better with the way nurses work than with the culture of medical practice, the latter described by one participant as “*a culture that you’re expected to know everything” (ID 4*.*1*, *director)* in which guidelines can be perceived as questioning or disrespecting their knowledge. Some participants indicated these differences in attitude are the result of how professions are educated, with medical staff being “*trained and programmed to work independently and make autonomous decisions [*…*]; whereas nursing staff are more used to protocols and working within a team (ID 18*.*1*, *director)”*. Most participants perceived guidelines unnecessary for senior staff with large clinical experience, however they were seen a *“useful aid memoir for the junior staff” (ID 22*.*2*, *doctor)*, particularly when the senior staff members are not around for escalation of questions e.g. during night-shifts. Because the level of senior staff in metropolitan hospitals is generally higher than in regional hospitals, it was suggested guidelines have more value in regional areas. Several respondents reflected on the fact that different stakeholders have different perceptions regarding whether a change is valuable, e.g. clinicians respond better to changes aiming to improve patient outcomes *(“We’re removing all emphasis on targets and concentrating on patient experiences*, *they [consultants] embrace [them] much better” ID 18*.*1*, *director)*.

Other influencing factors were the changes being in line with existing practices, which is related to the importance for hospitals of having the opportunity to adapt the intervention to the local context and resources (potential for reinvention) *(“If something is generated by a body like yourselves*, *and we don’t agree with it […]*, *or it doesn’t fit with our practice*, *[…] we might end up modifying it” ID 10*.*1*, *doctor)*. In the ED, the majority of patients need to be admitted or migrated out within certain timeframes, and bed availability in the hospital or elsewhere is often a limiting factor. This brings some challenges to managing mTBI patients because they have low acuity triage categories and generally spend longer time in the waiting room. Therefore, some respondents indicated that, ideally a pathway should also contain information on safe decision-making in less than ideal situations *(“A waiting room nurse may have ten-plus patients in the waiting room*. *By the time she sees this patient with a mild head injury it’s a long time and if there was any significant change or deterioration*, *it hasn’t been detected […]*. *So it’s pretty big responsibility” ID 4*.*3*, *nurse)*. Further, several participants indicated that because of the time pressures in the ED it is imperative that a new tool or practice is as quick, simple and clear as possible (low complexity), and does not lead to additional paperwork. Some participants mentioned that a pathway ideally should cross organisational boundaries so that, for example, there is an understanding between ward and ED with regard to the criteria for admission to the ward. However, the added complexity of arranging buy-in from other specialist groups reduces the likelihood and/or pace of implementation, as groups are likely to have different views.

It was considered very important that the changes made are actually visible (observability) to enhance and maintain motivation for change *(“Most staff do gain satisfaction from knowing that they’ve done a good job and have actually made a difference to someone’s lives*, *and they can see that they’ve made a difference” ID 6*.*1*, *director)*. Particularly doctors emphasised that strength of the evidence underpinning the recommended practices was seen as a prerequisite for any changes. In addition, participants referred to the necessity for the change to have a clear, unambiguous advantage over current practices. For instance, participants indicated that if a new tool would be implemented, it needed to have consequences in terms of subsequent management *(“So if one staff member performs the rule or test and then indicates the finding to another staff member*, *does that mean anything in terms of what might happen for the patient” ID 19*.*5*, *doctor)*. Also, given the ED manages a wide range of clinical presentations, a plethora of guidelines apply, which brings challenges in terms of awareness, access and application. It was perceived that the ED environment is often too hectic to go and find guidelines, particularly, if they aren’t immediately available at the point-of-care. Finally, some respondents reflected on the importance of any change coming with resources, or the need for it to be self-sustaining.

### System readiness for innovation

Overall, responses were mixed regarding whether participants perceived the management of patients with mTBI to be a priority for change for their respective EDs and whether they felt this topic aligned with their organisational priorities. Participants indicated priorisation typically occurs using criteria such as the clinical risk involved to patients, potential consequences for the organisation of “*getting it wrong*” *(ID 24*. *5*, *doctor)*, how common the condition is, and “*ease of fixing” (ID 3*.*2*, *doctor)* while keeping in mind the need to balance to resources. Several participants indicated that because mTBI is a common presentation to EDs (high volume) affecting many young people (e.g. sports injuries or assaults) it should be seen as a priority. However, although mild head injuries can lead to serious outcomes, in an environment dealing with many critically ill patients, it is seen as a relatively low risk condition, with low chances of poor outcomes *(“When it falls at the minor end of the scale and falls outside needing admission*, *there tends to be a falling down of the process in ED*. *We relax and say*, *this is a GP [general practitioner] thing*. *Get him out of here because we’ve got someone else coming in” ID 25*.*2*, *nurse)*. This means the focus is on acute complications such as the detection of intracranial lesions *(“We’re more concerned about missing the bleed than managing the mild [consequences]” ID 10*.*2*, *doctor)*. Although some participants indicated they thought there was certainly variation in this ‘acute part of management’, they generally considered it to be in line with what people would expect, and would be done elsewhere (low change commitment). So, in general, there was a relatively low tension for change with regard to managing acute symptoms.

In addition, there was mixed tension for change with regard to managing long term consequences. Several participants indicated that they thought “*the post effects of mild traumatic brain injury are significantly underestimated in EDs across the country” (ID 24*.*5*, *doctor)* and therefore that the biggest scope for improvement in this patient group would be in managing longer-term symptoms and follow-up (high change commitment). However, there were some concerns around this being outside the realm of the ED and whether the ED had the tools and resources to facilitate follow-up, and where to send patients (low change efficacy) *(“Nobody's interested in them and that includes us too […]*. *There's nothing really in place for them” ID 22*.*2*, *doctor)*. Several respondents, however, recognised that the ED could improve patient information, particularly with regard to educating patients around what to expect after discharge *(“I don’t think we fully emphasise the fact that they may still in six months [time] have some memory impairment or some difficulty concentrating or be a bit labile*. *And we don’t tell them that’s normal” ID 18*.*1*, *director)*.

Finally, respondents indicated they felt quality-focused indicators of mTBI management would not necessarily be in line with current accountability measures (see outer context).

### Implementation processes

Several aspects of existing and successful change management practices were discussed. In general, different professions have separate systems in place to organise and communicate changes within their own networks *(“Certainly the nursing staff have a sort of rigid program*, *and if they’re wanting to introduce something it has to go before a quality assurance council and it has to be endorsed and then it gets disseminated*. *Whereas medical*, *it’s more flexible and less rigid*, *and probably less effective”ID 18*.*1*, *director)*. Partly this seems a result of rostering difficulties *(“On the whole*, *most organisations have separate training times for nursing and medical [staff]*. *Our times are completely incompatible” ID 22*.*5*, *nurse)*. However, more evident in responses was that influence tends to be typically stronger within social networks, rather than across, particularly in medical staff *(“Nursing staff would probably be more open to respond to either/or*, *but the medical staff certainly would respond better to a medical lead than a nursing or allied health lead” ID 18*.*1*, *director)*. Participants often stressed the importance of involving both senior nursing and medical leaders (multidisciplinary leadership) to drive the changes top-down. The importance of their role and ‘leading by example’ (modelling) was often stressed. Indeed, choice of champion had proven to be a make-or-break factor in previous experiences *(“I think because the clinical champion lived in one particular person who didn’t have a lot of influence*, *it essentially fell on its feet” ID 6*.*1*, *director)*. One participant reflected on the importance of identifying the informal leaders, rather than the ones with “*the title in front of their names”* (ID 4.1, director).

Some difficulties in disseminating information arise because of the high staff turnover rates, particularly of doctors *(“I think the medical staff would probably be a bit less organised*, *in that we would tend to send an email out to everyone*, *some of [which] juniors*, *who*, *you know*, *the emails would bounce back because their inbox is full*, *they never check their work email; they don’t even know how to access it” ID 10*.*1*, *doctor)*, as well as difficulties in rostering within professions due to shift work (“*So say it’s February and we run a series of in-services and medical training*. *I’d say at least*, *probably*, *you need to have it staggered over about a month to catch additional people” ID 22*.*5*, *nurse)*. Because of these challenges, it was suggested to use the *“stable factors” (ID 37*.*2*, *nurse)* (i.e. consultants and nurses in metropolitan hospitals, and predominantly nurses in regional hospitals), as well as multi-channel communication processes.

### System antecedents for innovation

#### Structural characteristics

High turnover rates of staff was mostly seen as hampering implementation processes because of the constant loss of tacit knowledge. However, this can also be a window of opportunity *(“Unfortunately our resident and intern staff are constantly changing so in two months’ time they’ll be a completely differently lot*. *[…] in a way*, *it has some advantages in that if you establish a new procedure then the new doctors will only ever be taught that because they won’t know any other way” ID 4*.*2*, *doctor)*. In addition, most respondents indicated they felt the ED was stretched, sometimes to its very limits (capacity problems / organisational slack), by increasing numbers of patients presenting to the ED over the years, and the added pressure of time-based measures.

#### Receptive context for change

Some participants discussed the context of the ED in relation to change in general. They perceived the ED setting to be quite responsive to change (positive organisational culture in relation to change), as compared to other organisational units or more senior specialities, characterised by one respondent as having a culture of *“it’s always done like this—so this is how it’s going to be done” (ID 25*.*6*, *doctor)*. Some participants referred to the EDs track record of implementing large changes in the last 15 years, such as the introduction of short stay units and treatment pathways (positive history of change), which was partly perceived to result from the people who run EDs being interested in patient safety and improving processes (leadership and vision), but also that the pressures in ED are such that people are open to changes that realise better outcomes *(“The culture and environment of EDs is such that doing things which actually make a difference is something most ED staff are interested in because it’s an environment where there isn’t much time and decisions do need to be made and*, *the burden of presentations to ED is just rising inextricably so there’s a fertile ground really for any sort of intervention that can help keep people healthy” ID 19*.*5*, *doctor)*.

#### Absorptive capacity for new knowledge, organisational learning and knowledge management

The very factors that create receptivity and tension for change prove to be challenging for team-based learning as well as patient management in general. Indeed, several respondents indicated their clinical management decisions, to a certain extent, are influenced by the constantly changing context *(“It partly depends on what else you’re actually doing […]*, *but in general I’m happy [for the nurse to decide whether the patient is safe for discharge] if I’ve seen the patient once and I know and I trust the nurse*. *If it’s a bank nurse that I don’t know and they haven’t had senior nurses involved and they say ‘It’s fine’ then I go back and assess them myself” ID 24*.*5*, *doctor)*. Several participants reflected on the consequences for team-based learning. In ED, heavily supervised teams are put in place. However, these systems can break down at night (when the consultants are on call) and in rural places. Further, one participant reflected on the difficulties in ensuring teams of people work well together (team structure / satisfaction with the team) *(“Certain pairings or mixes of staff end up with the department not working at its best*. *[…] so it's balancing out the needs of us to recruit junior staff*, *providing a good mix of people who can teach versus those who can work and so it can get difficult”*, *ID 22*.*1*, *director)*. In addition, some participants indicated that a stretched environment is not conducive to learning and reflection. One ED director indicated that paying staff to attend education sessions may help to overcome this.

In addition, because work in the ED is very episodic, in general there is a lack of monitoring and feedback, which was perceived by one ED director to lead to a *“fairly defensive” (ID 6*.*1*, *director)* attitude to discussing problems. Some participants indicated that this culture is being changed *(“They’re trying to change that culture; that it’s not about dobbing*. *It’s not about picking on you*. *It’s about doing our best” ID 25*.*2*, *nurse)*. This lack of routine feedback appears to also contribute to more reactive approaches to problem solving; most participants indicated that change in their organisation was predominantly following adverse events or near misses rather than proactively planned *(“I think there’s a lot of reactivity that goes on but that’s just out of human habit; something crops up as a problem and you try to fix it*, *whereas there’s plenty of ways in which you can design change based upon future predictions or upon a perceived need rather than an actual event” ID 19*.*5*, *doctor)*. The presence or absence of IT systems in place was identified as an importance factor influencing this. For instance, one participant explained how their risk-management system helped decide whether a ‘near miss’ would require making ‘single loop changes’ (first level learning; i.e. try to prevent something happening again) or ‘double loop changes’ (second level learning; i.e. making changes to the system in order to prevent it happening again) (*“My quality improvement process goes like this […]*. *So the inbox comes from complaints*, *-top end complaints*, *as well*. *[…] It then gets filtered*, *and we decide from there*, *is it an individual that needs their behaviour changed*? *Or is it a process*?*” ID 24*.*1*, *director)*. Although participants stressed the importance of providing feedback on results of change efforts, and therefore having data to inform this, they indicated that, currently, the systems to support change are *“not great at doing that*, *in terms of it’s still very manual*, *so it’s very*, *very time consuming” (ID 18*.*1*, *director)*. This also seemed to limit opportunities to line up information derived from various hospital quality management systems in place (e.g. using real cases from morbidity and mortality meetings in continuous education sessions).

### Outer context

Cross-unit power and influence were discussed in terms of some of the tensions that arise from being a junior specialty at the entry point of the hospital, and seeing a broad range of patients. With a high proportion of patients generally requiring admission, to a certain extent, the ED management of patients is determined by what the specialist groups require if the patient were to be admitted under them *(“We’re not actually the gold standard voice*. *[…] So we have to deal with a lot of craft groups” ID 25*.*6*, *doctor)*. However, participants perceived that views of different groups around what constitutes high quality care do not always concur *(“[Radiology] looked at a normal CT scan as a failure of clinical decision making whereas we said that’s probably not how we look at it” ID 22*.*2*, *doctor)*. This is particularly problematic if there are no agreed cross-unit pathways because it is unclear under what circumstances patients can be admitted and therefore (particularly junior) staff might find it hard to argue the patient should be admitted *(“Generally we’d prefer someone who’s not quite right in that sense [amnesic] after a reasonable period of observation here to be admitted under the surgeons*. *But the surgical registrars in particular have decided discomfort about dealing with anything to do with the brain*. *They just say*, *“We have no expertise*, *” and […] the neurosurgeons say*, *“There’s no blood*. *There’s nothing we’re going to do*, *so deal with it” ID 25*.*5*, *doctor)*. Because the system appears characterised by unit level autonomy and accountability, overarching (e.g. CEO) leadership may be needed for cross-unit changes.

Further, there was a perception that organisational priorities and the accompanying accountability metrics (alignment with broader organisational performance metrics) are not generally driven by quality *(“Well*, *you have to be honest that at the moment we’re very much driven by exec and exec they’re following finance KPI’s*, *time and finance*. *They’re not following quality KPI’s in any shape or form” ID 25*.*1*, *director)*. Finally, financial systems focus on local costs (resources, trade-off) rather than appreciating whole of system costs of suboptimal care—i.e. across the continuum from acute care to within the community (lack of overall systems view / integration of systems) *(“the issue’s not about that the patient is going home in four hours*, *the issue is that the patient is actually returning to activities of daily living within two weeks rather than five weeks and the cost to public health”*, *ID 6*.*1*, *director)*. This may hinder patient-centered pathways, covering the whole patient journey, including follow-up if needed.

## Discussion

Several organisational factors were identified that will influence the design and delivery of our intervention. Tension for change was relatively low for managing acute mild head injury symptoms, and mixed for managing longer-term symptoms. Regarding successful change management practices, the importance of (visible) senior leadership for all professions involved was identified as a critical factor, as influence appears to be stronger within professions than across professions and particularly medical staff were more likely to accept change from a medical lead. This finding is in line with a study looking at social and geographical boundaries around senior nurse and physician leaders, which found the tendency to seek advice or discuss professional matters with those similar to themselves (‘professional homophily’) is stronger in medical leaders than senior nurses [72]. With regard to characteristics of the intervention the most important factor was whether the intervention was perceived as being in line with values and needs, with nurses being more accepting of guideline-based recommendations and tools than doctors. These findings confirm results of earlier studies [73,74]. For example, a study looking at attitudes towards guidelines of doctors and nurses working together in surgical teams [74] found that doctors prefer to follow the unwritten rules of medical practice, whereas nurses viewed guideline adherence as synonymous with professionalism. In addition, we found differences between ED doctors and specialists with regards to beliefs about what constitutes ‘high quality care’. This is particularly relevant for change in the ED, given its position as the entry-point of the hospital. So, although various professional groups both within and outside the ED are involved in the treatment of patients with head injuries, rather than sharing values and beliefs, all these hold the collective values of the group in which they have been socialised. Indeed, this has been shown to be a barrier for interprofessional collaboration [75,76] and spread of change [77], as shown in case studies in larger organisational settings, where stifling of change across boundaries between different professional groups was found because of the lack of shared work experience and shared belief systems [78]. The challenges this poses for the ED staff when cross-unit pathway or protocols are not in place (e.g. in negotiating the conditions under which specialists at wards (e.g. neurosurgeons) are willing to admit patients under their care), have also been described in an ethnographic study in two Australian EDs, which discusses the ‘tactics’ ED staff use in these negotiations, such as framing a case in such a way that it increases the chances the specialist will agree the patient needs their attention [79]. Finally, an unpredictable and hectic environment brings challenges in creating an environment in which team-based and organisational learning can thrive. High staff turnover causes a constant loss of tacit knowledge, and staff operate in constantly changing teams due to rotations and shift work (system antecedents for innovation). This was also identified as an important influencing factor in a study looking at adoption of spacers in paediatric emergency departments [15].

Although many of the factors we identified have been described in the literature, our interviews have given us valuable insight into the relative importance of them in different situations in relation to the management of patients with head injuries. It is important to remember though, that it is hard to predict how various factors will play out in a particular complex situation (e.g. synergistic effects between two seemingly unimportant barriers, turning them into an important obstacle to change) [28,80]. We have, a priori, chosen theories we considered likely to be relevant, and have used the model of diffusion in service organisations to guide the analysis and organise our results. Some other studies have used this model (or adapted versions) to explain adoption of innovations retrospectively in other hospital settings (e.g. [28,81,82]. These studies concluded the model was an effective analytical tool to explain variation in practice. It has been recommended to use the model in future trials—both prospectively and retrospectively, and in combination with (social) psychological theories [28]–which is planned for in our study. Diffusion theory highlights the importance of attributes of the innovation and the presence of intermediary actors such as opinion leaders for successful adoption and implementation. Although we had a good fit with the model with regards to those aspects, what also emerged from the data, however, was the importance of understanding how existing routine practices are being formed, defined and acted upon (or the ways the unwritten rules are formed), how they are held together (e.g. by trust relations between groups), and how they are threatened (for example by capacity problems). Therefore, theories on professions and communities of practice or theories concerning the relationship between individuals such as social network theory may have complemented our approach. One theory designed to take a more holistic approach, aiming to also understand how interventions are made workable and integrated in daily practice is the normalisation process theory [83]. By applying frameworks in different settings and studies, we will expand the understanding about its utility.

Some of the salient factors we identified in this study are potentially modifiable (mediators) and will therefore inform our intervention, resulting in intervention components targeting those factors. For instance, the results suggest that a lack of clinical leaders in each professional group ‘championing’ the new tools and providing an example for other staff is a barrier to staff using these tools. Hence we recruited both medical and nursing opinion leaders to lead local workshops, taking into account the characteristics suggested in the literature regarding their traits [51]. Similarly, limited slack resources can be overcome (at least for the trial period) by providing some reimbursement and communicating this in the early phases of recruitment. Some of the factors identified are outside the scope of what can be feasibly addressed in an improvement project like ours. For instance, participants stated that a new mTBI pathway would have most value if it covers the entire patient journey, or it at least clarifies agreements between relevant units involved within the hospital (e.g. wards and radiology). However, this requires up front agreement between units, which is likely to be much harder to achieve than within ED agreement only, because of the within unit accountability systems and the differences in views as to what would represent ‘high quality care’ between different professional groups. This led us to decide to communicate the practices of interest, but leave the EDs to decide whether to create or adapt a pathway, although ED staff were encouraged to develop pathways following positive experiences with the implementation to secure the changes made. In addition, we organised stakeholder meetings at the start of the intervention period and encouraged the ED staff involved in intervention delivery to invite non-ED stakeholders to these meetings, to provide opportunities for creating trust. Indeed, active participation of all professional groups and inclusion of the new device into a pathway or protocol proved to be strategies of early-adopters in a previous study [15].

Other (non-modifiable) factors (moderators) have practical implications that may need to be considered to ensure the intervention elements are a good fit with the ED environment (e.g. influencing modes of delivery or duration of intervention elements, or different content for different professional groups). For example, the high turnover rate of staff can be considered by designing brief educational sessions that can be repeated often and fitted within existing processes.

The inclusion of measures of factors in a process evaluation (e.g. inclusion of a local opinion leaders scale [84] in self-reported surveys completed by staff as well as inclusion of impressions of staff of the opinion leaders (factors such as credibility and availability) in qualitative semi-structured interviews post-intervention delivery) will enable testing of hypotheses about the extent to which different factors influence intervention effects. In addition, the findings of this paper help us understand the context in which these change processes take place and will aid interpretation of the trial results [85,86]. They complement the findings of an earlier paper [32] in which we examined the factors influencing the use of each of the four separate recommended practices (Table 1)–as perceived by ED clinicians, using a theoretical framework grounded in mainly individual behaviour change theory [87]. By tailoring our intervention to address the individual and organisational factors described in these two papers, we hope to maximise its effectiveness. The development and testing of the resulting intervention as part of a cluster randomised trial is reported separately [11,88].

This study has limitations. Only a subset of interviews was double coded for subthemes and factors (25%), however, the interpretation of the non-double coded data was checked by a second author (SB). Further, the factors we explored are self-reported, and therefore reflect factors perceived to be important influences. Triangulation with other methods to strengthen the validity of the results or quantify the relative importance of factors was outside the scope of our project. Also, our sample was limited to those within the ED and did not include other hospital decision makers such as CEO’s or directors of Medical Services, or clinicians working on wards. A wider sample could potentially have led to additional insights. Finally, due to the small numbers of sites, it was not possible to draw conclusions from the data with respect to potential differences according to factors such as size and rurality. We did however, have a wide range of variety in our sample with respect to those factors.

### Conclusions

We identified in a structured way a set of organisational pressures unique to the ED, that will help inform intervention elements, help ensure that the implementation processes are a good fit with the ED environment, and provide a rationale for selecting items for formative evaluation of the intervention. Our findings clearly show the importance of taking into account the social environment in which ED staff operate including the work relations with other professional groups.

## Supporting Information

S1 File Interview guide. (DOCX)

S2 File Tables with representative quotations. (DOCX)

## Abbreviations

CEO: Chief Executive Officer
ED: Emergency Department
(m)TBI: (mild) Traumatic Brain Injury
NET: Neurotrauma Evidence Translation
KPI: Key Performance Indicator

## References

[1] Maas AI, Stocchetti N, Bullock R. Moderate and severe traumatic brain injury in adults. Lancet Neurol. 2008; 7(8):728–741. 10.1016/S1474-4422(08)70164-9 18635021

[2] Abelson-Mitchell N. Epidemiology and prevention of head injuries: literature review. J Clin Nurs. 2008; 17(1):46–57. 18088259 10.1111/j.1365-2702.2007.01941.x

[3] Jagoda AS, Bazarian JJ, Bruns JJ Jr., Cantrill SV, Gean AD, Howard PK, et al Clinical policy: neuroimaging and decisionmaking in adult mild traumatic brain injury in the acute setting. Ann EmergMed. 2008; 52(6):714–748. 10.1016/j.annemergmed.2008.08.021 19027497

[4] Alves W, Macciocchi S, Barth J. Postconcussive symptoms after uncomplicated mild head injury. J Head Trauma Rehabil. 1993; 8:48–59.

[5] Ponsford J, Cameron P, Fitzgerald M, Grant M, Mikocka-Walus A. Long-Term Outcomes after Uncomplicated Mild Traumatic Brain Injury: A Comparison with Trauma Controls. J Neurotrauma. 2011; 28(6):937–946. 10.1089/neu.2010.1516 21410321

[6] Tavender EJ, Bosch M, Green S, O'Connor D, Pitt V, Phillips K, et al Quality and consistency of guidelines for the management of mild traumatic brain injury in the emergency department. Acad Emerg Med. 2011; 18(8):880–889. 10.1111/j.1553-2712.2011.01134.x 21843224

[7] Bazarian JJ, McClung J, Cheng YT, Flesher W, Schneider SM. Emergency department management of mild traumatic brain injury in the USA. Emerg Med J. 2005; 22(7):473–477. 15983080 10.1136/emj.2004.019273 PMC1726852

[8] Heskestad B, Baardsen R, Helseth E, Ingebrigtsen T. Guideline compliance in management of minimal, mild, and moderate head injury: high frequency of noncompliance among individual physicians despite strong guideline support from clinical leaders. J Trauma. 2008; 65(6):1309–1313. 19077619 10.1097/TA.0b013e31815e40cd

[9] Stuart B, Mandleco B, Wilshaw R, Beckstrand RL, Heaston S. Mild traumatic brain injury: are ED providers identifying which patients are at risk? J Emerg Nurs. 2012; 38(5):435–442. 10.1016/j.jen.2011.04.006 21774974

[10] Green SE, Bosch M, McKenzie JE, O'Connor DA, Tavender EJ, Bragge P, et al Improving the care of people with traumatic brain injury through the Neurotrauma Evidence Translation (NET) program: protocol for a program of research. Implement Sci. 2012; 7:74 10.1186/1748-5908-7-74 22866892 PMC3543324

[11] Bosch M, McKenzie J, Mortimer D, Tavender E, Francis J, Brennan S, et al Implementing evidence-based recommended practices for the management of patients with mild traumatic brain injuries in Australian emergency care departments: study protocol for a cluster randomised controlled trial. Trials. 2014; 15(1):281. 25012235 10.1186/1745-6215-15-281 PMC4107995

[12] Baker R, Camosso-Stefinovic J, Gillies C, Shaw EJ, Cheater F, Flottorp S, et al Tailored interventions to overcome identified barriers to change: effects on professional practice and health care outcomes. The Cochrane database of systematic reviews. 2010;(3):CD005470.12. 20238340 10.1002/14651858.CD005470.pub2 PMC4164371

[13] Grol R, Grimshaw J. From best evidence to best practice: effective implementation of change in patients' care. Lancet. 2003; 362(9391):1225–1230. 14568747 10.1016/S0140-6736(03)14546-1

[14] Greenhalgh T, Robert G, Macfarlane F, Bate P, Kyriakidou O. Diffusion of Innovations in Service Organizations: Systematic Review and Recommendations. Milbank Q. 2004; 82(4):581–629. 15595944 10.1111/j.0887-378X.2004.00325.x PMC2690184

[15] Scott S, Osmond M, O'Leary K, Graham I, Grimshaw J, Klassen T. Barriers and supports to implementation of MDI/spacer use in nine Canadian pediatric emergency departments: a qualitative study. Implement Sci. 2009; 4:65 10.1186/1748-5908-4-65 19828086 PMC2766417

[16] Greenfield D, Nugus P, Travaglia J, Braithwaite J. Factors that shape the development of interprofessional improvement initiatives in health organisations. Qual Saf Health Care. 2011; 20(4):332–337. 10.1136/bmjqs.2010.044545 21228438

[17] French B, Thomas LH, Baker P, Burton CR, Pennington L, Roddam H. What can management theories offer evidence-based practice? A comparative analysis of measurement tools for organisational context. Implement Sci. 2009; 4:28 10.1186/1748-5908-4-28 19454008 PMC2694144

[18] Kitson AL. The need for systems change: reflections on knowledge translation and organizational change. J Adv Nurs. 2009; 65(1):217–228. 10.1111/j.1365-2648.2008.04864.x 19032518

[19] Ferlie EB, Shortell SM. Improving the quality of health care in the United Kingdom and the United States: a framework for change. Milbank Q. 2001; 79(2):281–315. 11439467 10.1111/1468-0009.00206 PMC2751188

[20] Foy R, Ovretveit J, Shekelle PG, Pronovost PJ, Taylor SL, Dy S, et al The role of theory in research to develop and evaluate the implementation of patient safety practices. BMJ Qual Saf. 2011;20(5):453–459. 10.1136/bmjqs.2010.047993 21317181

[21] Meurer WJ, Majersik JJ, Frederiksen SM, Kade AM, Sandretto AM, Scott PA. Provider perceptions of barriers to the emergency use of tPA for acute ischemic stroke: a qualitative study. BMC Emerg Med. 2011;11–5. 21548943 10.1186/1471-227X-11-5 PMC3112102

[22] Musacchio NS, Gehani S, Garofalo R. Emergency department management of adolescents with urinary complaints: missed opportunities. J Adolesc Health. 2009;44(1):81–3. 10.1016/j.jadohealth.2008.05.011 19101462

[23] Pham JC, Kelen GD, Pronovost PJ. National study on the quality of emergency department care in the treatment of acute myocardial infarction and pneumonia. Acad Emerg Med. 2007;14(10):856–63. 17898249 10.1197/j.aem.2007.06.035

[24] Roy PM, Meyer G, Vielle B, Le Gall C, Verschuren F, Carpentier F, et al Appropriateness of diagnostic management and outcomes of suspected pulmonary embolism. Ann Intern Med. 2006;144(3):157–64. 16461959 10.7326/0003-4819-144-3-200602070-00003

[25] Grilli R, Lomas J. Evaluating the message: the relationship between compliance rate and the subject of a practice guideline. Medical Care. 1994;32(3):202–13. 8145598 10.1097/00005650-199403000-00002

[26] Compton S, Lang E, Richardson TM, Hess E, Green J, Meurer W, et al Knowledge translation consensus conference: research methods. Acad Emerg Med. 2007; 14(11):991–995. 17967960 10.1197/j.aem.2007.06.031

[27] Boudreaux ED, Cydulka R, Bock B, Borrelli B, Bernstein SL. Conceptual models of health behavior: research in the emergency care settings. Acad Emerg Med. 2009;16(11):1120–3. 10.1111/j.1553-2712.2009.00543.x 20053231 PMC5103302

[28] McMullen H, Griffiths C, Leber W, Greenhalgh T. Explaining high and low performers in complex intervention trials: a new model based on diffusion of innovations theory. Trials. 2015;16(1):242 26026849 10.1186/s13063-015-0755-5 PMC4465492

[29] Croskerry P. The feedback sanction. Acad Emerg Med. 2000, 7(11):1232–1238. 11073471 10.1111/j.1553-2712.2000.tb00468.x

[30] Holroyd BR, Bullard MJ, Graham TA, Rowe BH. Decision support technology in knowledge translation. Acad Emerg Med. 2007;14(11):942–948. 17766733 10.1197/j.aem.2007.06.023

[31] Curran J, Dartnell J, Magee K, Sinclair D, McGrath P. Organisational and professional interventions to promote the uptake of evidence in emergency care: effects on professional practice and health outcomes. Cochrane Database Syst Rev 2007(2):CD006557.

[32] Tavender E, Bosch M, Gruen R, Green S, Knott J, Francis J, et al Understanding practice: the factors that influence management of mild traumatic brain injury in the emergency department—a qualitative study using the Theoretical Domains Framework. Implement Sci. 2014;9(8). 10.1186/1748-5908-9-8 PMC3895840 24418161

[33] The Improved Clinical Effectiveness through Behavioural Research Group (ICEBeRG). Designing theoretically-informed implementation interventions. Implement Sci. 2006;1:4 16722571 10.1186/1748-5908-1-4 PMC1436012

[34] Sandelowski M. Combining qualitative and quantitative sampling, data collection, and analysis techniques in mixed-method studies. Res Nurs Health. 2000, 23(3):246–255. 10871540 10.1002/1098-240x(200006)23:3<246::aid-nur9>3.0.co;2-h

[35] Ryu WH, Feinstein A, Colantonio A, Streiner DL, Dawson D. Regional variability in the use of CT for patients with suspected mild traumatic brain injury. Can J Neurol Sci. 2009; 36(1):42–46. 19294887 10.1017/s0317167100006296

[36] Australian Bureau of Statistics. 1216.0 Australian Statistical Geography Standard (ASGS). July 2011.

[37] Ashford AJ. Behavioural change in professional practice: supporting the development of effective implementation strategies. Newcastle upon Tyne: Centre for Health Services Research; 1998.

[38] Grol RP, Bosch MC, Hulscher ME, Eccles MP, Wensing M. Planning and studying improvement in patient care: the use of theoretical perspectives. Milbank Q. 2007;85(1):93–138. 17319808 10.1111/j.1468-0009.2007.00478.x PMC2690312

[39] Kitson A, Harvey G, McCormack B. Enabling the implementation of evidence based practice: a conceptual framework. Qual Health Care. 1998;7(3):149–158. 10185141 10.1136/qshc.7.3.149 PMC2483604

[40] Wensing M, Bosch M, Foy R, Van der Weijden T, Eccles M, Grol R. Factors in theories on behaviour change to guide implementation and quality improvement in healthcare. Nijmegen: Centre for Quality of Care Research (WOK); 2005.

[41] Rogers E (ed.): Diffusion of Innovations, 5 edn New York Free Press; 2003.

[42] Foy R, MacLennan G, Grimshaw J, Penney G, Campbell M, Grol R. Attributes of clinical recommendations that influence change in practice following audit and feedback. J Clin Epidemiol. 2002;55(7):717–722. 12160920 10.1016/s0895-4356(02)00403-1

[43] Holt DT, Helfrich CD, Hall CG, Weiner BJ. Are you ready? How health professionals can comprehensively conceptualize readiness for change. J Gen Intern Med. 2010;25 Suppl 1:50–55. 10.1007/s11606-009-1112-8 20077152 PMC2806967

[44] Klein KJ, Sorra JS. The challenge of innovation implementation. Academy of Management Review. 1996;21(4):1055–1080.

[45] Weiner BJ, Belden CM, Bergmire DM, Johnston M. The meaning and measurement of implementation climate. Implement Sci. 2011;6:78 10.1186/1748-5908-6-78 21781328 PMC3224582

[46] Ovretveit J. Effective leadership of improvement: the research. Int J Clin Leader. 2008; 16:9.

[47] Ovretveit J. Leading improvement effectively. London: The Health Foundation; 2009.

[48] Baker DP, Day R, Salas E. Teamwork as an essential component of high-reliability organizations. Health Serv Res. 2006;41(4 Pt 2):1576–1598. 16898980 10.1111/j.1475-6773.2006.00566.x PMC1955345

[49] Lemieux-Charles L, McGuire WL. What do we know about health care team effectiveness? A review of the literature. Med Care Res Rev;2006, 63(3):263–300. 16651394 10.1177/1077558706287003

[50] Ham C. Improving the performance of health services: the role of clinical leadership. Lancet. 2003;361(9373):1978–1980. 12801754 10.1016/S0140-6736(03)13593-3

[51] Flodgren G, Parmelli E, Doumit G, Gattellari M, O'Brien MA, Grimshaw J, et al Local opinion leaders: effects on professional practice and health care outcomes. Cochrane Database Syst Rev. 2011(8):CD000125 10.1002/14651858.CD000125.pub4 21833939 PMC4172331

[52] Gershon RR, Stone PW, Bakken S, Larson E. Measurement of organizational culture and climate in healthcare. J Nurs Adm. 2004;34(1):33–40. 14737033 10.1097/00005110-200401000-00008

[53] Scott T, Mannion R, Marshall M, Davies H. Does organisational culture influence health care performance? A review of the evidence. J Health Serv Res Policy. 2003;8(2):105–117. 12820673 10.1258/135581903321466085

[54] Damanpour F: Organizational Innovations. A Meta-Analysis of Effects of Determinants and Moderators. Acad Manage J. 1991;34:555–590.

[55] Scott WR. Innovation in medical care organizations. A synthetic review. Med Care Rev. 1990;47:165–192. 10106771 10.1177/107755879004700203

[56] Yang B. The construct of the learning organization: dimensions, measurement, and validation. HRDQ. 2004;15(1):23.

[57] Ortenblad A. A typology of the idea of learning organization. Manage Learn. 2002;33(2):213–230.

[58] Garavelli AC, Gorgoglione M, Scozzi B. Managing knowledge transfer by knowledge technologies. Technovation. 2002;22(5):269–279.

[59] Armenakis AA. Making change permanent: a model for institutionalizing change interventions In: Research in Organizational Development and Change. Edited by Pasmore WA, Woodman RW. CT: JAI Press; 1999: 97–128.

[60] Nevis EC, Dibella AJ, Gould JM. Understanding Organizations as Learning-Systems. Sloan Manage Rev. 1995;36(2):73–85.

[61] Anderson N, West M. Measuring climate for work group innovation: development and validation of the team climate inventory. JOB. 1998;19(3):23.

[62] Rousseau V, Aube C, Savoie A. Teamwork Behaviors: A Review and an Integration of Frameworks. Small Group Research 2006;37(5):540–570.

[63] Casalino L, Gillies RR, Shortell SM, Schmittdiel JA, Bodenheimer T, Robinson JC, et al External incentives, information technology, and organized processes to improve health care quality for patients with chronic diseases. JAMA. 2003;289(4):434–441. 12533122 10.1001/jama.289.4.434

[64] Frambach RT, Schillewaert N. Organizational innovation adoption—A multi-level framework of determinants and opportunities for future research. JBR. 2002;55(2):163–176.

[65] Stetler CB, McQueen L, Demakis J, Mittman BS. An organizational framework and strategic implementation for system-level change to enhance research-based practice: QUERI Series. Implement Sci. 2008;3:30 10.1186/1748-5908-3-30 18510750 PMC2430586

[66] Gruen RL, Gabbe BJ, Stelfox HT, Cameron PA. Indicators of the quality of trauma care and the performance of trauma systems. Br J Surg. 2012;99 Suppl 1:97–104. 10.1002/bjs.7754 22441862

[67] Chassin MR, Loeb JM, Schmaltz SP, Wachter RM. Accountability measures—using measurement to promote quality improvement. N Engl J Med. 2010;363(7):683–688. 10.1056/NEJMsb1002320 20573915

[68] Chaix-Couturier C, Durand-Zaleski I, Jolly D, Durieux P. Effects of financial incentives on medical practice: results from a systematic review of the literature and methodological issues. Int J Qual Health Care. 2000;12(2):133–142. 10830670 10.1093/intqhc/12.2.133

[69] Kuper A, Lingard L, Levinson W. Critically appraising qualitative research. BMJ. 2008;337:a1035 10.1136/bmj.a1035 18687726

[70] Buetow S. Thematic analysis and its reconceptualization as 'saliency analysis'. J Health Serv Res Policy. 2010;15(2):123–125. 10.1258/jhsrp.2009.009081 19762883

[71] Francis JJ, Johnston M, Robertson C, Glidewell L, Entwistle V, Eccles MP, et al What is an adequate sample size? Operationalising data saturation for theory-based interview studies. Psychol Health. 2009;1–17. 20204937 10.1080/08870440903194015

[72] West E, Barron D. Social and geographical boundaries around senior nurse and physician leaders: an application of social network analysis. CJNR. 2005;37(3):132–148. 16268093

[73] Quiros D, Lin S, Larson EL. Attitudes toward practice guidelines among intensive care unit personnel: a cross-sectional anonymous survey. Heart & lung. 2007;36(4):287–297. 17628198 10.1016/j.hrtlng.2006.08.005 PMC2034210

[74] McDonald R, Waring J, Harrison S, Walshe K, Boaden R. Rules and guidelines in clinical practice: a qualitative study in operating theatres of doctors' and nurses' views. Qual Saf Health Care. 2005 8;14(4):290–294. 16076795 10.1136/qshc.2005.013912 PMC1744048

[75] Zwarenstein M, Reeves S. Knowledge translation and interprofessional collaboration: Where the rubber of evidence-based care hits the road of teamwork. J Contin Educ Health Prof. 2006;26(1):46–54. 16557506 10.1002/chp.50

[76] Muntlin A, Carlsson M, Gunningberg L. Barriers to change hindering quality improvement: The reality of emergency care. JEN. 2010 7;36(4):317–323. 10.1016/j.jen.2009.09.003 20624564

[77] West E, Barron DN, Dowsett J, Newton JN. Hierarchies and cliques in the social networks of health care professionals: implications for the design of dissemination strategies. Social Sci & Med. 1999;48(5):633–646. 10.1016/s0277-9536(98)00361-x 10080364

[78] Ferlie E, Fitzgerald L, Wood M, Hawkins C. The nonspread of innovations: the mediating role of professionals. Acad Manage J. 2005;48(1):18.

[79] Nugus P, Bridges J, Braithwaite J. Selling patients. BMJ. 2009;339:b5201 20008442 10.1136/bmj.b5201

[80] Nilsen P. Making sense of implementation theories, models and frameworks. Implement Sci. 2015;10:53 10.1186/s13012-015-0242-0 25895742 PMC4406164

[81] Dalrymple PW, Lehmann HP, Roderer NK, Streiff MB. Applying evidence in practice: a qualitative case study of the factors affecting residents' decisions. Health Inform J. 2010;16(3):177–188. 10.1177/1460458210377469 20889848

[82] Makowsky M, Guirguis L, Hughes C, Sadowski C, Yuksel N. Factors influencing pharmacists' adoption of prescribing: qualitative application of the diffusion of innovations theory. Implement Sci. 2013;8(1):109. 24034176 10.1186/1748-5908-8-109 PMC3847669

[83] Murray E, Treweek S, Pope C, MacFarlane A, Ballini L, Dowrick C, et al Normalisation process theory: a framework for developing, evaluating and implementing complex interventions. BMC medicine. 2010;8:63 10.1186/1741-7015-8-63 20961442 PMC2978112

[84] Helfrich CD, Li YF, Sharp ND, Sales AE. Organizational readiness to change assessment (ORCA): development of an instrument based on the Promoting Action on Research in Health Services (PARIHS) framework. Implement Sci. 2009;4:38 10.1186/1748-5908-4-38 19594942 PMC2716295

[85] Helfrich CD, Damschroder LJ, Hagedorn HJ, Daggett GS, Sahay A, Ritchie M, et al A critical synthesis of literature on the Promoting Action on Research Implementation in Health Services (PARIHS) framework. Implement Sci. 2010;5(1):82. 20973988 10.1186/1748-5908-5-82 PMC2988065

[86] Rycroft-Malone J, Seers K, Chandler J, Hawkes CA, Crichton N, Allen C, et al The role of evidence, context, and facilitation in an implementation trial: implications for the development of the PARIHS framework. Implement Sci. 2013;8(28). 10.1186/1748-5908-8-28 PMC3636004 23497438

[87] Michie S, Johnston M, Abraham C, Lawton R, Parker D, Walker A. Making psychological theory useful for implementing evidence based practice: a consensus approach. Qual Saf Health Care. 2005;14(1):26–33. 15692000 10.1136/qshc.2004.011155 PMC1743963

[88] Tavender EJ, Bosch M, Gruen RL, Green SE, Michie S, Brennan SE, et al Developing a targeted, theory-informed implementation intervention using two theoretical frameworks to address health professional and organisational factors: a case study to improve the management of mild traumatic brain injury in the emergency department. Implemt Sci. 2015;10(1):74. 10.1186/s13012-015-0264-7 PMC4446082 26003785

