# Supplementary material for: The Many Organisational Factors Relevant to Planning Change in Emergency Care Departments: A Qualitative Study to Inform a Cluster Randomised Controlled Trial Aiming to Improve the Management of Patients with Mild Traumatic Brain Injuries
Source: PLoS One. 2016 Feb 4;11(2):e0148091. doi: 10.1371/journal.pone.0148091 (PMC4742078; doi:10.1371/journal.pone.0148091)
Supplement: S1 File — (DOCX) [file pone.0148091.s001.docx]

**S1 Additional file: Interview guide**

**ED Directors**

|  | **Domain** | | | | |
| --- | --- | --- | --- | --- | --- |
| **Questions / prompts** | **1** | **2** | **3** | **4** | **5** |
| Can you give us an idea about the human resources that are available for the management of patients with mTBI in this ED?   - Which professions would be involved, approximate number of staff for each profession - What do you think about their availability for managing patients with mTBI in this ED? - Eg availability of team members during out of hours? Differences day / night shift - How are teams organised? |  |  |  | **✓** |  |
| What about physical resources?   - Eg CT scanner on site? - Do you have a short-stay unit? If so-number of beds? Protocol for mTBI? What is the procedure when the short stay unit is full? |  |  |  | **✓** |  |
| What type of medical record system is in use?   - Paper based / electronic? Use of reminders/prompts/pathways in system? - Do doctor and nurse notes ‘talk to each other’? |  |  |  | **✓** |  |
| Can you tell me about the work environment in the ED in relation to quality improvement / change management?   - What would you tell new staff about how ‘things are’ in the ED? - What are the unwritten rules that relate to changing practice in the ED? |  |  |  | **✓** |  |
| In the ED you see a wide range of conditions with varying severity, do you see improving the management of mTBI as a priority for your ED?   - How are ‘topics’ prioritised? Staff involved? Bottom-up / top-down? - What would be the incentives for you / your ED to participate in a KT/QI project on the management of mTBI / in general? |  | **✓** |  |  |  |
| Has your ED been involved in quality improvement projects in the past, either internally or externally initiated?   - Can you briefly describe some examples? Any previous activities in head injury? - What turned out to be the key lessons from these experiences? - What about the communication and education tools used? |  |  | **✓** |  |  |
| How do changes generally get implemented?   - Change agent/champion? Clinical leadership? - Do you have a person whose specific role is to manage the quality improvement process? |  |  | **✓** |  |  |
| Who decides upon the work processes / changes in the ED?   - Would staff generally be involved? - Who decides upon what tools or methods to use for, say, risk assessment in patients with mTBI? - What processes are in place if change processes cross units? |  |  | **✓** |  |  |
| Are you aware of any guidelines used for the management of patients with mTBI?   - Do you have guidelines for the management of patients with mTBI / head injury? - Any protocols / tools in place for assessing risk and need for CT scan, any tools for assessing PTA? - Are they externally / internally developed? | **✓** |  |  |  |  |
| Can you tell us about the use of guidelines / protocols / policies in general in the ED?   - What do you think about the use of these tools in the ED setting? - When would they have a higher chance of getting used? | **✓** |  |  |  |  |
| What, if any, processes are in place for feedback?   - Any routinely collected data? Individual/team/departmental level? |  |  |  | **✓** |  |
| Often, a strong incentive for making changes is when things go (almost) wrong. What systems, if any, are in place to learn from mistakes?   - (blame-free) reporting systems? Feedback? - Organisation-wide systems? Linked in with each other? - Discussion of events with people from other ward / organisation? - Would underlying processes be questioned / redesigned if needed? |  |  |  | **✓** |  |
| Are there organisational or legal regulations that influence how things are done in the ED in general and for the management of mTBI specifically?   - Eg the implementation of the 4-hour rule how will this affect the management of this type of patient? - Regulations, resources, … |  |  |  |  | **✓** |
| Any other matters that may be relevant in managing patients with mTBI that has not been mentioned yet? |  |  |  |  |  |

1: The innovation; 2: System readiness for innovation; 3: Implementation processes; 4: System antecedents for innovation; 5: Outer context

**ED Staff**

|  | **Domain** | | | | |
| --- | --- | --- | --- | --- | --- |
| **Questions / prompts** | **1** | **2** | **3** | **4** | **5** |
| Are you aware of guidelines for the management of patients with mTBI?   - How credible are the sources or developers of these guidelines? - Are you familiar with the recommendations in these guidelines for assessment of risk, referral for imaging and the provision of patient information? | **✓** |  |  |  |  |
| Can you tell us about the use of guidelines / protocols / policies in general in the ED?   - What do you think about the use of these tools in the ED setting? | **✓** |  |  |  |  |
| Say someone new starts in your ED, how do they get to know how this hospital manages patients with mTBI?   - What would you tell the new person about the work environment in this ED in relation to quality? What are the unwritten rules? How would you describe the culture of the ED? |  |  |  | **✓** |  |
| What –if any– processes are in place for feedback?   - In what instances would feedback be provided? Individual/team/departmental level? |  |  |  | **✓** |  |
| How do changes generally get implemented? Previous experiences? Important lessons? What works best in the ED?   - Change agent/champion? Clinical leadership? Who needs to be involved? Who takes decisions? - What communication channels or strategies? Reminders? |  |  | **✓** |  |  |
| In the ED you see a wide range of conditions with varying severity, do you see improving the management of mTBI as a priority for your ED?   - If there was one thing you could change in your hospital to improve the management of mTBI in ED what would you change? - How are ‘topics’ prioritised? |  | **✓** |  |  |  |
| Do staff suggest changes? Do you feel free to? Are staff involved in thinking about changes needed? |  |  |  | **✓** |  |
| How do team members communicate with each other over the management of mTBI?   - Does that work well? Doctor – nurse coordination / communication? Medical record, face-to-face, LAN-paging, …. - Do you feel team roles are clear? Have you ever been trained in how to work together in providing quality care? |  |  |  | **✓** |  |

1: The innovation; 2: System readiness for innovation; 3: Implementation processes; 4: System antecedents for innovation; 5: Outer context
