# Supplementary material for: The Many Organisational Factors Relevant to Planning Change in Emergency Care Departments: A Qualitative Study to Inform a Cluster Randomised Controlled Trial Aiming to Improve the Management of Patients with Mild Traumatic Brain Injuries
Source: PLoS One. 2016 Feb 4;11(2):e0148091. doi: 10.1371/journal.pone.0148091 (PMC4742078; doi:10.1371/journal.pone.0148091)
Supplement: S2 File — (DOCX) [file pone.0148091.s002.docx]

**S2 Additional file: Tables with representative quotations**

**The innovation**

| **Themes** | **Influencing factors** | **Representative quotations*** |
| --- | --- | --- |
| Perceived innovation characteristics | *Compatibility with values and needs – guidelines/protocols fit better with nursing practice than medical culture* | “…particularly medical staff who are generally taught to think outside the square a little bit and I think there’s a culture that you’re expected to know everything. Which means that if you’re looking something up then you can’t know it therefore you’re almost admitting that you’re not as smart as you think you are… and I think that’s something that needs to be overcome.” |
|  |  | “Because in a lot of medical opinion, a pathway is monkey-see, monkey-do and trying to get them to agree to use a pathway can be quite difficult. So, the nurses do use them but once again you’re sort of pushing it uphill against a doctor who is not really that interested in it.” |
|  |  | “But pathway driven nursing seems to work really well. Particularly when you’re trying to push a lot of stuff back onto the nurses and have a lot of stuff nurse driven.“ |
|  |  | “Medical staff are actually trained and programmed to work independently and make autonomous decisions, and be independent, so therefore it doesn’t apply to them. So, they’re often quite resistant to seeing a global change like this... Whereas nursing staff are more used to protocols and nursing staff are more working within a team. Whereas medical staff are actually, you know, …you go through university, you’ve been taught to be fully autonomous, and then you’re being told, “Hey, hang on. You have to actually adhere by someone else’s rules rather than your own.” As they get more senior they get less flexible.” |
|  | *Compatibility with values and needs – varies for different stakeholders* | “…you know a lot of these targets, they’re targets and… we’re removing all emphasis on targets and concentrating on patient experiences, they [consultants] embrace [them] much better. That’s what they think they’re here for, that’s what they want to do: they want to make a difference to the patient… they don’t want to necessarily meet a target that’s decided from someone in the Health Department who has never worked in an Emergency Department.“ |
|  |  | “It’s important that it’s a, ideally a clinically driven change not seen to be purely a KPI driven change or a management requirement change, but is actually something that’s going to benefit the patient… is going to be something that has a true impact.” |
|  | *Being in line with existing practices* | “…and it doesn’t alter or interfere too much with what else they’re doing.” |
|  | *Potential for reinvention* | “So, the problem is, if something like that is generated by a body like yourselves, and we don’t agree with it, that’s when there is a problem. Or that it doesn’t fit with our practice, we might look at that and say, we wouldn’t scan, and there’d be 10%, 20% extra people we’d be scanning if we followed that guideline. So we might end up modifying it.” |
|  | *Pathway needs to accommodate various possible scenario’s / be applicable in all situations* | “…but the reality is that more and more of these patients are spending one to two hours in and around the ED before they’re getting a medical assessment done. I mean, that obviously works well for the patients that are in the bays. That’s a bit more tricky to apply that to patients in the waiting room. But that’s probably where there needs to be some discrimination around who goes in and who doesn’t. And also what you do with people that should go in, but there’s no bay……and in the grand scheme of things they don’t get one, and how you actually manage those patients in the waiting room. I mean that’s, that’s not unique to here, but I think that’s a generic sort of challenge.” |
|  |  | “A waiting room nurse may have ten-plus patients out in the waiting room. By the time she sees this patient with a mild head injury it’s a long time and if there was any significant change or deterioration, it hasn’t been detected until they’re actually seen by that waiting room nurse. So it’s pretty big responsibility.” |
|  |  | “I think one of the most challenging things is getting the doctors to see these patients within the triage category time. Sometimes, these patients, they should be seen within an hour … that are, you know, been waiting two, three hours plus. So, it really is important then that the nursing staff have been observing and making sure these patients are okay and the GCS is, is you know, stable……and, and hopefully that they’re noting any sort of change … in their condition or their behaviour.” |
|  | *Low complexity- easy to use* | “There’s no point in implementing something that’s a 20 page turner where … it’s the easiness, the use of those guidelines has to be relatively easy. …If something flows, it’s much easier to implement than something that requires a lot of stop and …” |
|  |  | “… some of them [guidelines] are not streamlined, they’re not like tick boxes and you know exactly what to do. Some of them are wordy. And I don’t think they lend themselves to our environment.” |
|  |  | “I think in terms of the patient process or pathway itself, it needs to be easy to use, it needs to be, preferably less paperwork. Something that doesn’t require them to actually do countless paperwork.” |
|  | *Complexity – cross unit change is ideal but much more complex* | “Ideally I think it would be something that would need to have the CEO’s support to translate it to the clinical units involved rather than just one particular unit. If you’re looking at just an audit of “Are you guys good at risk stratifying mTBI?” maybe that might be fine within the ED, but then it doesn’t translate to a continuum of care.” |
|  |  | “I think there has to be recognition that it is an issue in emergency as well as hospital wide. So I think there’s so many issues across this, you know, involvement of rehabilitation services or a referral pathway. It’s a hard one… “ |
|  |  | “Then you will have to involve the hospital processes [names specific stakeholder units and groups in the hospital]. Now the time frame for implementing that sort of change immediately is altered to a much more prolonged process. Is it more acceptable? If there’s a hospital wide process – probably. Is it more accountable? Yes. Uh, but I think … you’d have to accept that the time frame for making that change would be …and perhaps it would be more diluted. I imagine any forum where you go to; there will be a diverse range of opinions. Of clinical practice. And trying to get any group to conform or agree on a process is hard. So I think you’re going to immediately dilute your message. Just by involving more people.” |
|  | *Observability of change results* | “Most staff do gain satisfaction from knowing that they’ve done a good job and have actually made a difference to someone’s lives, and they can see that they’ve made a difference.” |
|  |  | “The feedback seems to have changed people in terms of if they know what they’re doing, if they know that they’re changing and it’s making a difference rather than, “Well, we think it’s making a difference, but we’re not sure.” Or, “It’s not making any difference and we should be doing something else.” |
|  | *Strength of the evidence – sufficient evidence = prerequisite* | “Yep, I guess it depends on what is the evidence…that it’s important to change what we’re doing now? And if there was compelling evidence, we’d make it a very high priority.” |
|  |  | “Because the nurses will go, “We’re doing this score.” And the docs will go, “What score?” And you go, “This score that we’ve just had education [in].” And they’ll go, “I do not believe in this. I’ve [got] to see randomised control trial evidence about this.” |
|  | *Clear, unambiguous advantage* | “Yeah, there’s plenty of protocols and guidelines and in reality they rarely get followed unless it suits a particular purpose, like getting the patient admitted or … getting what you want for the patient. Because they’re all there for the patients: we’re not trying to be subversive. We’re just, most people are trying to get on with their job. And so … to gain compliance with a protocol guideline there usually has to be some sort of outcome that people realise that that’s a good thing …And it’s, yeah, immediately obvious that it’s just going to add value.” |
|  |  | “I think there has to be some measure of evidence based medicine that you know actually makes a difference. I think that would probably be the most important thing in terms of trying to make a change.” |
|  | *Plethora of guidelines apply in ED – info overload* | “That’s the problem with guidelines and protocols and things like this. And there’s hundreds of them, and I’m not aware of them…because it’s not the way we work. Particularly in ED […]. You can’t tell me before you start your job here you have to read 250 guidelines.” |
|  |  | “I don’t know, there’s so many protocols, guidelines, check lists, this, that and this, and it becomes pretty mind numbing at times.” |
|  | *Environment too busy/hectic to search for them* | “so there’s all this information for people and when you’re on the floor and there’s 20 people waiting and you’re being pressured to do things……to then go and try and find a guideline on the internet doesn’t always happen.” |
|  |  | “And our experience with guidelines, like most emergency departments, is that everyone’s too busy to look at guidelines… they’re great in an offline and in an educative forum and you can develop them and educate people as much as you can… but in the real setting on – on the floor the only thing that really helps is actually good supervision and good clinical leadership on the floor.” |
|  | *CEO support* | “…can we reproduce this in local centres with executive support? Can it be self-sustaining, and at what cost is that going to occur?” |
|  | *Resources - reimbursement* | “So I think, it really is important to recognise that if they are doing more work what’s in it for them. I think to do more work and obviously without resources it’s always difficult, considering that we all have enough work to do.” |

**System readiness for innovation**

| **Themes** | **Influencing factors** | **Representative quotes** |
| --- | --- | --- |
| *System readiness / tension for change* | *Organisational priority setting processes* | “I think anything high volume, high risk gets prioritised over other things. Any problem that is exceptionally high risk, even if it’s low volume tends to probably get the next priority. And then it depends on what the risk to the patient is; what the risk to the department is; what the risk to the hospital and health service is if you get it wrong.” |
|  |  | “You know you make it [mTBI] a priority once you’ve marketed it to me it’s a real problem. So at the moment how much of a problem do I think it is? I don’t think it’s much of a problem. I don’t know it’s much of a problem. I’m sure if I really think about it it’s much more of a problem than we think about. I certainly know people who’ve had minor head injuries who are really not functioning months and even year afterwards. So if I step back from day to day madness I’m sure there could be an economic argument, there could be a huge, to lift its priority ... but personally for me at the moment, real low.” |
|  |  | “It’s always related to risk benefit analysis. Of the resources. And of the conditions that we see, so … we don't miss a lot of intracranial bleeds, but a miss – that can have high consequence for the patient.” |
|  |  | “… I mean there’s a combination of things like the size of the problem for us; the ease of fixing it; and the strength of evidence behind it, they’d be the main things. And also what people want because, you know, you can’t get people to do things that they don’t feel about.” |
|  |  | “Something that’s going to put patients at risk, um, that obviously gets prioritised first. So it depends on how patient impact and how important it’s going to be.” |
|  | *High volume condition* | “… again it probably just all comes down to clinical relevance and your patient mix. If it’s something that we see a lot of…” |
|  |  | “Oh, it’s common. So that, I mean we deal mostly in common things. Although severity is what you see on the TV shows on emergency medicine. But the vast majority of emergency medicine is common things and it would be probably in the top 10 or 15 things with chest infections, and low back pain, and broken hips.” |
|  | *Potentially high risk – but generally perceived as low risk in environment dealing with life threatening situations* | “The vast majority are fine but it’s about identifying the needle in the haystack really and making sure that you don’t miss the ones with the red flags.” |
|  |  | “When it falls at the minor end of the scale and falls outside needing admission, we relax and say, this is a GP thing. Get him out of here because we’ve got someone else coming in. And the pressure’s off. “ |
|  |  | “Uh, I mean, we have a lot of issues in emergency department. Everything that turns up is an issue, so I know that, that we’re more concerned about missing the bleed than managing the mild [consequences].” |
|  | *Current management acute symptoms perceived acceptable* | “These patients as I say they’re not… they just tend to be churned through fairly satisfactorily without too many hiccups.” |
|  |  | “Well, I think it would be more seen that because it’s a mild injury…that it’s something that they’re okay and comfortable with, and there is not a lot that they could do differently that would make any difference […] Our current management within the ED…I think is okay. And I think the management within nearly every ED would be fairly similar…” |
|  | *Relatively high change commitment - Underestimation of longer term symptoms* | “Well, the guideline on the internet is obviously going to be talk about major head injuries and intubating people and stabilising their necks and all the other traumatic stuff and so the minors, … it will be in the guideline but it will be the last paragraph, “and by the way these people can end up having their lives ruined by this.” |
|  |  | “I guess one of the first things perhaps is obviously the recognition and the awareness factor, firstly in an organisation, perhaps isn’t a lot of perception about not only what is considered TBI, what assessment of TBI is, but also the morbidity related to it afterwards as well. So it’s certainly not, certainly not in my organisation, a fairly high priority type clinical issue in the department, and maybe because it’s seen as, from the context of not necessarily requiring hospital admission, and that patients are quite often, they’re episodic but they’re not followed up, so there’s not a lot of awareness about that. And I think that’s changing to some extent.” |
|  |  | “I suppose it’s a reasonably important priority. It’s up there with a lot of other things. … I think it is something that’s neglected a little bit… it’s something a lot of people don’t realise that if you, if you get knocked out briefly, you may well have, you know, problems for a couple of weeks with headaches ... and difficulty calculating and short-term memory loss and feeling irritable and cognitive dysfunction in other ways. So, it can have a fair effect on somebody’s life for a couple of weeks after that.” |
|  |  | “I don’t think we put as big a priority on it as we should. Like we just sort of think if someone’s had a little knock to the head, or a little LOC for a little time, they’ll be alright. We sort of look at more a longer period of LOC as a priority. So it’s not a big priority. It should be, but it’s not.” |
|  | *Low change efficacy – no resources or outside realm* | “So the treatment now is quite focused on identifying the patients with intracranial lesions but not that much identifying the patients who might have issues later on. Because if from a, strictly speaking from a, you know, the ED realm You don’t think about that aspect. It’s all about, do they – do they need something now and urgently.” |
|  |  | “… we're not aware of what else we might be doing for them. So there's people where you wonder how much anterograde and retrograde amnesia they’ve got. Have they got post-concussion syndrome, their headache's still going on after six weeks, you know, there's nothing really in place for them.” |
|  |  | “I don’t think you can identify at the bedside who’s going to have the post-concussive [symptoms]… I don’t think there’s a good tool.” |
|  | *Importance of advice* | “But also because there’s probably a degree of unrecognised complications, for this patient group. So perhaps we could, give better discharge advice at the time of the first discharge if we know what the common problems are that they come back with at their plan review.” |
|  |  | “I don’t think we fully emphasise the fact that they may still in six months [time] have some memory impairment or some difficulty concentrating or be a bit labile. And we don’t tell them that’s normal, that can happen, and what to do about it.” |
|  | *Alignment with organisational priorities - Management driven priorities very time-based (also see outer setting)* | “In terms of what is being measured from an organisational point of view, I mean particularly if you’re saying “Well the issue’s not about that patient actually going home in four hours, that issue is about that patient actually returning to activities of daily living within two weeks rather than five weeks and the cost to public health is X versus that.” That’d be nice. But that’s not how we work and … I mean that’s the task that all public health practitioners, including emergency physicians, face is that things are measured, the hard things that are measured are not necessarily the ones that matter.” |
|  |  | “Probably depends who you talk to. Efficiency for the ED is a big outcome measure. So the main key performance indicators that ED is reporting on is, times and percentages and times to treatment or from treatment, and percentages of people who don’t wait, a percentage of bypassable; percentage of failed admissions.” |

**Implementation processes**

| **Themes** | **Influencing factors** | **Representative quotes** |
| --- | --- | --- |
| *Existing systems for dissemination* | *Different systems within professional silos* | “Certainly the nursing staff have a sort of rigid programme, and if they’re wanting to introduce something it has to go before a quality assurance council and it has to be endorsed and then it gets disseminated. Whereas medical, it’s more flexible and less rigid, and probably less effective, to be honest.” |
|  | *Rosters don’t line up* | “On the whole, most organisations have separate training times for nursing and medical [staff]. Our times are completely incompatible.” |
| *Influence & power* | *Social networks - Influence* | “Medical staff tend to listen a bit more to doctors. So if you’ve got a neurologist or a neurosurgeon who go into bat for you guys, as a new tool, absolutely. Just because it tends to give it more weight. And I think that’s doctors everywhere. So it does ... I think, any kind of education, especially if it’s going to be more targeted, like, the actual pointy end, as opposed to the general background of it all, you’d be better off probably trying to get at least doctors or senior nursing staff to educate medical staff.” |
|  |  | “The medical staff probably would respond better to a medical lead, and the nursing staff would probably be more open to respond to either/or. But the medical staff certainly would respond better to a medical lead than a nursing lead or an allied health lead.” |
|  |  | “I think because the clinical champion lived in one particular person who didn’t have a lot of influence, it essentially fell on its feet.” |
|  |  | “So you would need a two-pronged attack. On the whole, if you want something done, engage the nurses and it will get done, if you engage them in the right way. But you need to make sure the medics understand what is going on.” |
|  |  | “And we would have clinical nurse champions; and clinical consultant champions…who would drive it with everyone. And would drive it across disciplines, whether it was medical or nursing…” |
|  |  | “I think if you can get agreement between the leaders both of medical and nursing in the department then you’ve got a much better chance of it filtering out to the rest of the troops.” |
|  | *Senior leadership - Start top-down* | “If it’s come from [a] senior, and so this is the way it’s going to be, whether some are reluctant, I think it will be implemented.” |
|  |  | “Look, it would have to start at the top with the consultants and the nurses in charge.” |
|  | *Informal leadership* | “… the thing is identifying the leaders because the leaders aren’t always the ones who have actually got the title in front of their name. There are other staff who, because of their natures and personalities, lead - not always in a positive way. So I guess it’s ensuring too that those people who have the potential to … sway opinion can be brought on board as well. And hopefully then they can contribute in a positive way rather than a negative way.” |
| *Considerations in current change management practices* | *Difficulties in implementing changes due to high turnover rates* | “… well, the, the nursing staff are very good at propagating information. So it would just need to go through the nursing leadership, and then they have a very effective way of that getting to every staff member[…] Their communication system is really very well organised. I think the medical staff would probably be a bit less organised, in that we’d probably, we would tend to send an email out to everyone, some of whom, some of the juniors who, you know, the emails would bounce back because their inbox is full, they never check their work email; they don’t even know how to access it. We’d have a teaching session, and two or three will be on night duty, one might have just decided not to come, you know, so people fall through the gaps with that. So … it needs to be multi-media, multi-source, so, didactic teaching, email, maybe even hard copy as well.” |
|  |  | “Because not all staff members have staff emails or access to computers and if they do check them.” |
|  | *difficulties in implementing changes due to shift work* | “I find scheduling stuff a major challenge People say, “Great idea,” but it doesn’t, doesn’t matter if you can only get say one out of every 10.” |
|  |  | “So say it’s February and we run a series of in-services and medical training. I’d say at least, probably, you need to have it staggered over about a month to catch additional people.” |
|  | *Multi-channel communication needed* | “We would use a multifaceted communication strategy. So, newsletter, communication book, group e-mails, word of mouth, education sessions. And I often tick a few of those boxes when I, just deciding how important a message is.” |
|  |  | “Emails are not great because people just delete them. Information sessions are not great because people miss them because they’re on nights. Brochures are not great because people don’t read them. One to one education’s not great because it takes forever to get through staff. Something stapled to your payslip is not great because already they do that too much and you just unstaple it and throw it away. And not everyone gets their payslip. Something that’s on the front page of the newspaper is probably the way to go.” |
|  |  | “If we wanted to ensure that everybody with a mild traumatic brain injury adhered to certain protocols then the stability of the department is the nurses. Otherwise you’d be running education sessions every three, every 12 weeks for the new interns. I mean because that’s where the consistency is, that’s your regular group of workers. So you would, say to the nurses, “Look, everybody who you think has a mild traumatic brain injury who meets this criteria needs a CAT scan.” So the nurses would know that, they would do the checklist, they would say to the medical staff, “Oh, look. We normally CT these people, here’s our checklist.” And they go, “Oh, okay. Yeah fine. Let’s scan them. For example.” |
|  |  | “I think particularly engaging with the nurses. I think nurses and allied health be very key in selling that message, because, as you know they’re usually the constant and the doctors are not. And they’re quite often the ones that are involved in terms of the, after initial assessment they’re involved in an ongoing care to some extent, or ongoing observations of them, so they’re probably more key in terms of actually picking up…” |

**System antecedents for innovation**

| **Themes** | **Influencing factors** | **Representative quotes** |
| --- | --- | --- |
| *Structural characteristics* | *High turn-over rates causes constant loss of tacit knowledge* | “It’s the turnover. I’ve just, the thing is HMOs [are] changing over 10 weeks. Registrars every 6 months. And consultants, who are constant, [are] not doing night shift. So you line those three up and it’s a massive, massive change.” |
|  |  | “Unfortunately, our resident and intern staff are constantly changing so in two months’ time they’ll be a completely differently lot than was there before. So I guess that, in a way, it has some advantages in that if you do establish a new procedure then the new doctors will only ever be taught that because they won’t know any other way.” |
|  | *Organisational slack in combination with time-based measures – capacity problems* | “So they [wards] have their own patients and they are looking after things long-term: they have a different set of pressures. I know they have clinics and all sorts of things that they have to run, but ED is, we can’t control our workload, we can’t, so we can’t schedule a clinic here or there. If there’s 100 patients that want to be seen right now then we have to see those 100 patients.“ |
|  |  | “I think one of the most challenging things is getting the doctors to see these patients within the, triage category time. … Sometimes you know, these patients … they should be seen within an hour that are … waiting two, three hours plus. … it’s then so important then that the nursing staff have been observing and making sure these patients are okay and the GCS is, is you know, stable …and, and hopefully that they’re, you know, they’re noting any sort of change … in their condition or their behaviour.” |
|  |  | “… some of the barriers are the fact that we’re very time based in terms of our measures. That and, not having the dedicated resource … for someone to just come in and say “I’ll do the assessment now”, rather than “Oh look I’m busy on the ward. I know it’s important but I have another two hours of work before I can assess this patient”, it’s just not tenable in a performance based emergency department. So that’s significant in terms of, in looking after those patients.” |
| *Receptive context for change* | *Positive culture in relation to change- as compared to other, more senior, specialties* | “I think, it’s a fairly good, I’ve, I haven’t worked many other areas: I’ve never worked in any other ED, but up on the wards, it’s very closed and everything’s done by, this is, “It’s always been done like this, so this is how it’s going to be done.” Whereas … we’re encouraged to, you know, bring new ideas in, …research-based practice…” |
|  |  | “The Emergency Department may be less [change aversive] because it’s more junior a speciality, so we’re always willing to embrace different ways of doing things: we have short-stay units…we have all sorts of clinical pathways, and streaming of patients, and things like that. So we’re, not as change averse as some of our other specialist colleagues, but I still think, yes, it requires an enormous amount of effort to implement even sometimes the simplest of changes in medical practice.” |
|  | *Organisational history of change – positive track record* | “It’s a biased perspective but ED’s have changed almost beyond recognition in the last 12 to 15 years. And my impression is that Emergency Departments especially in Victoria have been very quick to adapt to significant practice changes in a vast array of areas. So whether it’s trauma or early management of sepsis or stroke or observation units, or treatment pathways. So my perception is that as an environment with the right strategy it’s a, an easy place to implement meaningful change.” |
|  |  | “There’s lots of reasons. It partly relates to clinical leadership in the area and the fact that the people who run ED’s are interested in patient safety and process improvement. But also that the culture and environment of EDs is such that doing things which actually make a difference is something most ED staff are interested in because it’s an environment where there isn’t much time and decisions do need to be made and, the burden of presentations to ED is just rising inextricably so there’s a fertile ground really for any sort of intervention that can help keep people healthy.” |
| *Absorptive capacity for new knowledge, organisational learning and knowledge management* | *Stretched environment not conducive for learning / reflection* | “…so many doctors just want to turn up and go home…, and particularly when you’ve got the hospital saying, “We don’t want to pay you overtime. We don’t want to do this. We don’t want to do that.” There’s not a lot of incentive to actually go the extra step… and actually change anything…” |
|  | *Constantly changing teams – clinical management depends on what’s going on and whether you know and trust staff members in your shift* | “… in general, particularly on weekends, which is when I’d see probably the most of this stuff, just because that’s when the risky things that people do that cause this happen…I tend to be reasonably busy and if the nurses come up to me and say, “Look, the neuro obs are fine after four hours.” It’s like that’s fine. I don’t need to see them again. […] It partly depends on what else you’re actually doing. So there’s some variation in that, but in general I’m happy [for the nurse to decide whether the patient is safe for discharge] if I’ve seen the patient once and I know and I trust the nurse. If it’s a bank nurse that I don’t know and they haven’t had senior nurses involved and they say ‘It’s fine’ then I go back and assess them myself […] realistically, what you want is a [consistent] risk management strategy, regardless of time of attendance or who sees them.” |
|  | *Constantly changing teams – challenges to team-based learning*  *Team structure*  *Team satisfaction* | “The other thing is obviously observational things that you get that certain pairings or mixes of staff end up with the department not working at its best. if you label Consultants I suppose as running a team, you'd say that when so and so's in charge of the team, that usually things get a bit out of control, but the staff get a lot of teaching from that person and a lot of supervision and support and they actually enjoy working with them, whereas somebody else has always got things under control but nobody likes working with them. [...]and so it's balancing out the needs of us to recruit junior staff, providing a good mix of people who can teach versus those who can work and so it can get difficult. How do you actually access all of these things together?” |
|  | *Lack of routine feedback / “punitive” culture* | “I think the natural tendency for some emergency departments to receive feedback is to be fairly defensive, or to celebrate the good but not actually consider the bad. And protect their staff from it.” |
|  |  | “I think people are afraid of upsetting other people and don’t want to feel like they’re being watched or that they’re being dobbed on by their colleagues. And they’re trying to change that culture; that it’s not about dobbing. It’s not about picking on you. It’s about doing our best.” |
|  |  | “I think the emergency department is a very episodic care department. We don’t get much feedback at all. I think that’s something that’s missing. Maybe some of the practitioners there don’t actually care too much about feedback, maybe that’s why they choose this speciality. I mean that’s quite possible.” |
|  | *Single-loop learning dominant* | “… we certainly do get the knee-jerk reaction sometimes when there’s something’s gone wrong. We think, “Oh, my god. We’ve got to change that. We’ve got to do it better.” |
|  |  | “I think there’s a lot of reactivity that goes on but that’s just out of human habit; something crops up as a problem and you try to fix it, whereas there’s plenty of ways in which you can design change based upon future predictions or upon a perceived need rather than an actual event.” |
|  |  | “I think that still emergency departments on a whole can do better. Their frameworks about how to measure their risk events and how to view it … I mean some places are very, very mature … but there’ll probably be a small percentage of those hospitals.” |
|  | *Double-loop learning* | ”My quality improvement goes like this […]. So the inbox comes from complaints, -top end complaints as well.[…] It then gets filtered, and then we decide from there, is it an individual that needs their behaviour changed? Or is it a process?” |
|  |  | “Look I think that the main thing is, as you say, that we look an opportunity when we get the feedback to actually say “Well what can we do with that information to see what we’ve done well, what we’ve done badly and change it, and try to systematise it so that actually that’s going to be a good thing all the time”. So that’s something that I feel is a very useful thing.” |
|  | *Supporting systems* | “Drive it [is key to arranging change], and ongoing feedback and ongoing auditing. And showing them what, what was happening in terms of the results that we were getting. So our system isn’t great at doing that, in terms of it’s still very manual, so it’s very, very time consuming.” |

**Outer context**

| **Themes** | **Influencing factors** | **Representative quotes** |
| --- | --- | --- |
| *Cross-unit / hospital level factors* | *Cross unit influence of other specialist groups* | “But that’s the very nature of ED is that we see, we’re often the entry point for the hospital and we see a very broad range of patients. Every craft group has their opinion about how they want the patients that end up coming under them managed.” |
|  |  | “I don’t think there’s a message from the management in….it varies between different groups of professionals. So emergency physicians would have a different threshold from radiologists, from neurosurgeons, from general surgeons. […] And so there’s quite a lot of variability between different working groups.” |
|  | *Different views of what would be ‘high quality care’ or best pathway* | “Then I went to radiology and they said, “You doctors will just use this as an excuse to scan everything. “Anyone who falls over with a head laceration, you’ll just want to scan them. You just want to protocolise everything.” And so I’ve had resistance from radiology.” |
|  |  | “I can recall the radiology department complaining about the number of normal CT scans that we had. Yeah they looked at a normal CT scan as a failure of clinical decision making whereas we said that’s probably not how we look at it.” |
|  | *No agreed cross-unit pathways* | “Generally we’d prefer someone who’s not quite right so to speak in that sense [amnesic] after a reasonable period of observation here to be admitted under the surgeons. But the surgical registrars in particular have decided discomfort about dealing with anything to do with the brain. They just say, “We have no expertise,” and, “What do the neurosurgeons say,” and well, the neurosurgeons say, “There’s no blood. There’s nothing we’re going to do, so deal with it.” |
|  | *CEO support needed for cross-unit pathways* | “Ideally I think it would be something that would need to have the CEO’s support to translate it to the clinical units involved rather than just one particular unit.” |
|  | *Financial incentives / focus on KPIs* | “Well, you have to be honest that at the moment we’re very much driven by exec and exec they’re following finance KPI’s, time and finance. They’re not following quality KPI’s in any shape or form.” |
|  |  | “There's a lot of concentration on KPI's at the moment. We seem to have no trouble meeting the KPI's with respect to seeing people within the appropriate timeframes across all the categories and the issues that we have are getting people out of here in four hours to go home and bring them to the ward in eight hours. So there's a lot of work going on around that and looking at the discharge process really. Discharge either to the ward or to home.” |
| *Factors related to the wider healthcare system* | *Unit accountability / financial incentives* | “ For a CT brain basically radiology are happy to do them and so because they don’t have to ask a radiology consultant they just have to give it to a technician, although the party line would be that the junior doctor should ask the consultant on the floor to get it, does that happen? No. Not a lot because if you haven’t got an ugly person to deal with in another department you don’t need to bother challenging and having a debate with your consultant who may disagree so you take the easy path. So as a junior doctor I just take the form, I take it round to radiology they do the test, that’s it. So in fact what actually happens is anyone can order a CT brain on anybody.[…] Really in reality they could be doing thousands of unnecessary, and probably are. And actually so are we and again the system is that the radiologists are happy they get $[amount]. It’s money for them. They’re paid. No one’s questioning it, nobody. It’s a big problem.” |
|  | *No (financial) system that covers entire pathway for patients if they need transfer / system capacity problems*  *System capacity problems* | “GPs are perhaps the ones that follow up these patients and actually see the morbidity or the costs of what actually happens to these patients. We only see the costs purely from an episodic point of care.” |
|  |  | “Wherever we’ll get someone to accept them. The most common place is [hospital] they tend to accept our patients in ED. My understanding is that their radiology staff are kicking up problems about us sending patients in because they don't feel that they should be doing our radiology. I think the cost exceeds their income for a CT out of hours.” |
|  |  | “The ED sees X number of patients of X conditions, but they can only accept about [percentage] at most, of the patients into the hospital, based on their clinical service profile. The rest of them need to be migrated out. So at the moment […] our capacity is stretched, clearly stretched to the limit.” |

* Quotes were edited for readability, however, no substantive changes were made. Text that has been added for clarity has been placed in brackets.
